# Role of Doppler Ultrasound in the Evaluation of Arteriovenous Fistula Maturation and Detection of Complications in Hemodialysis Patients

**DOI:** 10.7759/cureus.84681

**Published:** 2025-05-23

**Authors:** Prama Polavarapu, Satish D Patil, Sandeep Patil, Santosh Patil

**Affiliations:** 1 Radiology, BLDE (Deemed to be University) Shri B. M. Patil Medical College, Hospital and Research Centre, Vijayapura, IND; 2 Radiodiagnosis, BLDE (Deemed to be University) Shri B. M. Patil Medical College, Hospital and Research Centre, Vijayapura, IND; 3 General Medicine, BLDE (Deemed to be University) Shri B. M. Patil Medical College, Hospital and Research Centre, Vijayapura, IND; 4 Urology, BLDE (Deemed to be University) Shri B. M. Patil Medical College, Hospital and Research Centre, Vijayapura, IND

**Keywords:** arteriovenous fistula, doppler ultrasound, hemodialysis, maturation, vascular access

## Abstract

Objective: This study aimed to assess the utility of Doppler ultrasound (DUS) in the preoperative planning, intraoperative guidance, and postoperative monitoring of arteriovenous fistulas (AVFs) for hemodialysis, focusing on vascular mapping, maturation assessment, and early complication detection.

Methodology: This prospective study included 109 patients undergoing AVF creation for hemodialysis access. Preoperative DUS was performed to assess the cephalic vein's and radial artery's diameters and flow parameters. Postoperative evaluations were conducted on days 1 and 7 and at four weeks to monitor vein diameter, flow rates, and complications. Patients were followed until successful cannulation or the determination of primary failure.

Results: Of the 109 patients, 63 (57.8%) were male, with a mean age of 52.4 years. Diabetes was present in 59 (54.1%) patients and hypertension in 57 (52.3%). The mean preoperative cephalic vein diameter was 1.98 ± 0.58 mm, increasing to 9.07 ± 0.75 mm at four weeks postoperatively. Fistula flow rates improved from 261.1 ± 106.3 mL/min on day 1 to 689.7 ± 138.09 mL/min at four weeks. By week 4, 56 (51.4%) AVFs had achieved adequate maturation. Primary failure occurred in 27 (24.8%) patients and was significantly associated with diabetes, hypertension, smaller preoperative vessel diameters, and lower four-week flow rates (p < 0.001). The mean time to maturation was 38.03 ± 6.8 days, and the mean time to first successful cannulation was 50.4 ± 8.1 days.

Conclusion: DUS parameters, particularly preoperative vessel diameters and four-week flow rates, significantly predict AVF maturation and primary failure. Routine ultrasound evaluation facilitates optimal vascular access planning, the early detection of complications, and improved AVF outcomes in hemodialysis patients.

## Introduction

Chronic kidney disease (CKD) represents a significant global health burden, with end-stage renal disease (ESRD) affecting millions of patients worldwide who require renal replacement therapy for survival. Among the available treatment modalities, hemodialysis remains the cornerstone, with approximately 70% of ESRD patients dependent on this life-sustaining procedure [1]. The success of hemodialysis critically depends on establishing and maintaining adequate vascular access, with the arteriovenous fistula (AVF) universally acknowledged as the preferred method due to its superior patency rates, lower infection risk, and reduced morbidity compared to other access types [2].

Creating a functional AVF involves surgical anastomosis between an artery and a vein, typically in the upper extremity, followed by a crucial maturation period. During this period, the vein undergoes significant hemodynamic and structural changes, including dilatation and wall thickening, to accommodate the increased blood flow required for effective dialysis [3].

Doppler ultrasound (DUS) has emerged as an invaluable tool in evaluating and monitoring AVFs, offering several advantages, including its non-invasive nature, absence of radiation exposure, and real-time imaging capabilities [4]. This modality provides detailed information on vessel anatomy, blood flow dynamics, and key parameters for assessing fistula maturation and identifying complications [5]. Early detection of such complications enables timely intervention, potentially preventing access failure and reducing the morbidity associated with vascular access dysfunction [6]. Routine surveillance using DUS has been shown to enhance AVF outcomes and reduce the need for emergency interventions [7]. This study aimed to assess the utility of DUS in the preoperative planning, intraoperative guidance, and postoperative monitoring of AVFs for hemodialysis, focusing on vascular mapping, maturation assessment, and early complication detection.

## Materials and methods

Study design and duration

This prospective study was conducted at BLDE (Deemed to be University), located in Karnataka, India, from August 2023 to December 2024 after obtaining approval from the Institutional Ethics Committee of BLDE (Deemed to be University) Shri B. M. Patil Medical College, Hospital and Research Centre (approval number: BLDE (DU)/IEC/942/2023-24). One hundred nine dialysis-dependent patients undergoing AVF creation for hemodialysis access were enrolled. Patients were followed from the time of fistula creation until either successful cannulation or the determination of primary failure.

Study population

The study population consisted of dialysis-dependent patients between 20 and 70 years old who had undergone primary radiocephalic AVF (RC-AVF) creation. Only those willing to participate in follow-up evaluations were included. Patients with vascular grafts or central venous catheters or those who declined participation were excluded. Written informed consent was obtained from all participants before the institutional review board granted inclusion and ethical approval.

Preoperative evaluation

All patients underwent a standardized preoperative DUS evaluation to assess the diameters, flow characteristics, and wall morphology of the radial artery and cephalic vein. These parameters were used to determine vascular suitability and assist in surgical AVF creation planning.

Ultrasound equipment and settings

Ultrasound imaging was performed using the GE VOLUSON S8 BT18 (GE HealthCare, Chicago, IL, USA) and the GE VERSANA PREMIER (GE HealthCare, Chicago, IL, USA). Both machines had high-frequency linear transducers capable of B-mode imaging at 7 MHz and Doppler imaging at 5 MHz. Examinations were conducted in a temperature-controlled environment using pre-warmed gel to minimize vasoconstriction and enhance patient comfort.

Sonographer training and standardization

All ultrasound scans were performed by certified vascular sonographers who had completed a structured training program in vascular access imaging. This imaging included a minimum of 20 supervised vascular scans, testing of interobserver reliability (with an intraclass correlation coefficient >0.85), and adherence to a standardized scanning protocol. Regular peer-review audits were conducted to ensure consistency and quality across all examinations.

Examination technique

Ultrasound assessments were conducted with patients in the supine position and slight trunk elevation to facilitate the visualization of upper extremity vasculature. Arterial evaluation was performed from the root of the arm toward the hand, while venous assessment was conducted from the periphery toward the thorax. Both transverse and longitudinal scanning planes were utilized. Each session included B-mode imaging for anatomical evaluation, color Doppler to assess flow patterns, spectral Doppler for velocity measurements, and volume flow calculations to assess vascular performance. Measurements were taken at three standardized points, and the average was used for analysis to improve reproducibility.

Surgical technique

AVF creation was performed by experienced vascular surgeons under local anesthesia using a standardized surgical technique. An end-to-side anastomosis of the cephalic vein to the radial artery was constructed using 6-0 polypropylene sutures under loupe magnification. The arteriotomy size was adjusted according to intraoperative vessel diameters, and intraoperative Doppler confirmed flow adequacy. The presence of a thrill and bruit was documented immediately after surgery.

Postoperative monitoring

DUS evaluations were repeated on the first postoperative day, the seventh day, and four weeks post-surgery. During these assessments, vein diameter, blood flow rate, and the presence of any complications were carefully monitored. A structured management protocol was followed: flow below 300 mL/min or absent thrill prompted immediate re-evaluation and possible surgical revision; hematomas or infections were assessed and treated accordingly; and symptoms suggestive of steal syndrome warranted further vascular evaluation and potential intervention.

AVF maturation assessment

The AVF's maturation was assessed at four weeks using the "Rule of 6" criteria, which included a blood flow volume of at least 600 mL/min, an outflow vein diameter greater than 6 mm, and sufficient vein depth to permit successful cannulation.

Flow volume calculation

Blood flow was measured in the brachial artery just above the elbow crease, where the vessel runs obliquely and provides a favorable insonation angle. Flow volume was calculated using area × mean velocity × 60. The Doppler angle was maintained at 60 degrees or less to ensure precision in velocity measurements.

Surveillance strategy

Postoperative surveillance focused on the early detection of fistula dysfunction. Flow rates between 700 and 1,300 mL/min were considered optimal, while values below 500 mL/min warranted further evaluation. Measurements below 300 mL/min were treated as indicators of imminent thrombosis and prompted immediate clinical intervention.

Quality assurance

All ultrasound examinations were performed by experienced sonographers who received standardized vascular access imaging training. Ultrasound systems were routinely calibrated according to manufacturer specifications to ensure measurement accuracy. Each parameter was measured three times, and the average of these readings was used for analysis to enhance reliability and reproducibility.

Statistical analysis

Data were compiled using Microsoft Excel (Microsoft Corp., Redmond, WA, USA) and analyzed with IBM SPSS Statistics for Windows, V. 21.0 (IBM Corp., Armonk, NY, USA). Quantitative variables were expressed as means, medians, standard deviations, and ranges. Qualitative variables were presented as frequencies and percentages. Comparisons of mean values were performed using the two-tailed Student's t-test, and a p-value of less than 0.05 was considered statistically significant.

## Results

A total of 109 patients underwent AVF creation. The majority were aged 41-60 years, with 62 (56.9%) patients, followed by 37 (33.9%) patients aged >60 years and 10 (9.2%) patients aged 20-40 years. There was a slight male predominance (63; 57.8%). Comorbidities included diabetes in 59 (54.1%) patients, hypertension in 57 (52.3%), and both in 27 (24.8%). The mean weight was 68.6 ± 13.9 kg. Laboratory findings showed a mean hemoglobin level of 10.5 ± 1.2 g/dL and serum creatinine of 8.6 ± 2.03 mg/dL. The average systolic and diastolic blood pressures were 140 ± 15.2 mmHg and 90 ± 10.04 mmHg, respectively. Preoperative DUS showed a mean radial artery diameter of 1.98 ± 0.63 mm and a flow volume of 42.2 ± 15.8 mL/min (Table 1).

**Table 1:** Distribution of patients according to age

Variables	Total number (N = 109)
Age (in years)	
20-40	10 (91.7%)
41-60	62 (56.9%)
>60	37 (33.9%)
Gender	
Male	63 (57.8%)
Female	46 (42.2%)
Comorbidities	
Diabetes	59 (54.1%)
Hypertension	57 (52.3%)
Diabetes + hypertension	27 (24.8%)
Weight (mean ± SD)	68.6 ± 13.9
Hemoglobin levels (g/dL)	10.5 ± 1.2
Systolic blood pressure (mean ± SD)	140 ± 15.2
Diastolic blood pressure (mean ± SD)	90 ± 10.04
Creatinine levels (mg/dL) (mean ± SD)	8.6 ± 2.03
Preoperative radial artery diameter (mm)	1.98 ± 0.63
Preoperative arterial flow (mL/min)	42.2 ± 15.8

Postoperative Doppler assessments showed that the cephalic vein diameter remained at 1.98 ± 0.58 mm on day 1, increasing to 7.45 ± 0.68 mm on day 7 and 9.07 ± 0.75 mm by week 4. Flow rates increased from 261.1 ± 106.3 mL/min on day 1 to 391.06 ± 124.3 mL/min on day 7 and to 689.7 ± 138.09 mL/min by week 4. No patients achieved maturation on day 1 or 7; however, by week 4, 56 (51.4%) AVFs had matured, while 53 (48.6%) remained immature (Table 2).

**Table 2:** Distribution of patients according to vein diameter

Variables	Postoperative day 1	Postoperative day 7	Postoperative week 4
Vein diameter (mm) (mean ± SD)	1.98 ± 0.58	7.45 ± 0.68	9.07 ± 0.75
Fistula flow rate	261.1 ± 106.3	391.06 ± 124.3	689.7 ± 138.09
Maturation status			
Adequate	0	0	56 (51.4%)
Inadequate	109 (100%)	109 (100%)	53 (48.6%)

The mean time to maturation was 38.03 ± 6.8 days, and the mean time to first successful cannulation was 50.4 ± 8.1 days (Table 3).

**Table 3:** Distribution of patients according to outcomes

Outcome	Time to maturation (days)	First successful cannulation (days)
Mean ± SD	38.03 ± 6.8	50.4 ± 8.1

Primary failure was significantly associated with diabetes, hypertension, and their combination (p < 0.001). Although more frequent in patients >60 years, age did not reach statistical significance (p = 0.16). The failure group had significantly smaller preoperative cephalic vein diameters (1.5 ± 0.27 mm vs. 2.5 ± 0.29 mm; p < 0.001), with similar trends persisting on postoperative day 1, day 7, and week 4. Radial artery diameter was also smaller in failed AVFs (1.45 ± 0.29 mm vs. 2.58 ± 0.27 mm; p < 0.001). Week 4 flow rates were lower in the failure group (615.6 ± 124.04 mL/min vs. 774.06 ± 100.1 mL/min; p < 0.001). Both time to maturation and time to first cannulation were significantly delayed in failed cases (p < 0.001) (Table 4).

**Table 4:** Association of primary failure with clinical and Doppler parameters An asterisk (*) indicates a p-value of less than 0.05, signifying statistical significance df: degrees of freedom; AVF: arteriovenous fistula

Parameter	Successful AVF (mean ± SD/n)	Primary failure (mean ± SD/n)	Test values	df	Effect size	P-value
Age >60	27 (24.7%)	10 (9.1%)	χ² = 1.9	1	Cramér's V = 0.11	0.16
Diabetes	40 (36.6%)	19 (17.4%)	χ² = 3.8	1	Cramér's V = 0.17	0.05
Hypertension	36 (33%)	21 (19.2%)	χ² = 9.8	1	Cramér's V = 0.26	0.002*
Diabetes + hypertension	7 (6.4%)	20 (18.3%)	χ² = 20.3	1	Cramér's V = 0.37	<0.001*
Preoperative vein diameter (mm)	2.5 ± 0.29	1.5 ± 0.27	t = 17.6	156	Cohen's d = 3.57	<0.001*
Postoperative day 1 vein (mm)	2.5 ± 0.29	1.5 ± 0.27	t = 17.6	156	Cohen's d = 3.57	<0.001*
Postoperative day 7 vein (mm)	7.41 ± 0.7	4.48 ± 0.67	t = 23.7	156	Cohen's d = 4.3	<0.001*
Postoperative week 4 vein (mm)	9 ± 0.72	5.13 ± 0.77	t = 28.5	156	Cohen's d = 5.4	<0.001*
Radial artery diameter (mm)	2.58 ± 0.27	1.45 ± 0.29	t = 21.6	156	Cohen's d = 4.1	<0.001*
Fistula flow (day 1) (mL/min)	228.8 ± 115.6	200 ± 121.8	t = 1.1	156	Cohen's d = 0.24	0.27
Fistula flow (day 7) (mL/min)	389.3 ± 129.2	350 ± 121.02	t = 1.39	156	Cohen's d = 0.31	0.167
Fistula flow (week 4) (mL/min)	774.06 ± 100.1	615.6 ± 124.04	t = 7.5	156	Cohen's d = 1.4	<0.001*
Time to maturation (days)	32.6 ± 6.4	42.7 ± 1.4	t = 9.9	156	Cohen's d = 2.1	<0.001*
First cannulation (days)	43.4 ± 6.6	56.5 ± 2.5	t = 11.1	156	Cohen's d = 2.3	<0.001*

Complications were significantly more common in failed AVFs: thrombosis occurred in seven (25.9%) vs. three (3.6%) cases (p < 0.001), stenosis in two (7.4%) vs. zero (0.0%) cases (p = 0.01), and infection in four (14.8%) vs. two (2.4%) cases (p = 0.01) (Table 5).

**Table 5:** Association of primary failure of AVF with complications A p-value of less than 0.05 was considered significant df: degrees of freedom; AVF: arteriovenous fistula

Complications	Primary failure		χ²	Effect size	df	P-value
	Yes	No				
Thrombosis	3 (3.6%)	7 (25.9%)	10.75	0.38	1	<0.001
Stenosis	0	2 (7.4%)	6.06	0.28	1	0.01
Infection	2 (2.4%)	4 (14.8%)	6.45	0.29	1	0.01

## Discussion

DUS has emerged as an essential, non-invasive tool for the preoperative evaluation, postoperative monitoring, and surveillance of AVFs. This study aimed to evaluate the role of DUS in assessing AVF maturation and predicting complications in patients undergoing hemodialysis. A total of 109 patients who underwent AVF creation for hemodialysis access were included. Male patients were predominant (57.8%), aligning with the findings of Siddiqui et al., who reported a male predominance of 63% in their cohort of ESRD patients [8]. Most patients (56.9%) were aged 41-60 years, followed by 33.9% over 60 and only 9.17% in the 20-40-year age group. This age distribution reflects the epidemiology of CKD, which primarily affects middle-aged and older adults due to the cumulative impact of long-standing comorbid conditions. Siddiqui et al. also reported a comparable mean age of 57 years in their study population [8].

Diabetes (54.1%) and hypertension (52.3%) were the most common comorbidities, with 24.8% of patients presenting with both. These findings are consistent with global trends, as diabetes and hypertension are leading contributors to the development of ESRD. Mendes et al. similarly observed diabetes in 45.8% and hypertension in 62.5% of their study population [9]. These comorbidities are particularly important, as they not only drive CKD progression but also adversely affect vascular health and thereby influence AVF outcomes.

The preoperative mean cephalic vein diameter was 1.98 ± 0.58 mm, which increased to 7.45 ± 0.68 mm by day 7 and 9.07 ± 0.75 mm at four weeks postoperatively. This progressive vein dilatation is a key indicator of successful AVF maturation. Robbin et al. reported that a vein diameter of ≥4 mm is associated with successful cannulation and AVF maturation [10]. Our findings confirm that significant vein expansion occurs within the first week following AVF creation, with continued dilatation over time. This pattern of vascular remodeling is essential for determining the optimal timing of initial cannulation.

The preoperative radial artery diameter was 1.98 ± 0.63 mm with a mean flow volume of 42.2 ± 15.8 mL/min. When stratified by risk, 32.1% of patients had normal flow, 58.7% had intermediate flow, and 9.2% demonstrated high-risk flow characteristics. These preoperative parameters are critical in determining the likelihood of AVF success. Jemcov reported that a radial artery diameter of <1.5 mm was associated with an increased risk of AVF failure [11], while Bashar et al. noted that an arterial flow volume of <40 mL/min predicted poor maturation outcomes [12]. Our results underscore the importance of detailed preoperative vascular assessment in predicting AVF patency and maturation. Smaller preoperative vein and artery diameters were significantly associated with primary failure, supporting individualized access planning. Ferring et al. found that preoperative ultrasound mapping increased the proportion of patients receiving AVFs and improved primary patency rates [13].

Postoperative flow rates progressively increased from 261.1 ± 106.3 mL/min on day 1 to 391.06 ± 124.3 mL/min on day 7 and 689.7 ± 138.09 mL/min by week 4. According to the Kidney Disease Outcomes Quality Initiative (KDOQI) guidelines, a flow rate >600 mL/min is considered adequate for dialysis [2]. Our findings demonstrate that by week 4, the mean flow exceeded this threshold, suggesting successful maturation in most cases. However, individual variability in flow patterns highlights the need for personalized monitoring. While early postoperative flow rates (day 1 and day 7) did not significantly differ between successful and failed AVFs, a marked difference was noted at week 4. Patients with successful AVFs had higher mean flow rates (774.06 ± 100.1 mL/min) compared to those with primary failure (615.6 ± 124.04 mL/min; p < 0.001). This suggests that while early flow measurements may be inconclusive, flow at four weeks is a reliable indicator of AVF maturation. Itoga et al. reported that 4-6 weeks post-creation flow measurements strongly predicted long-term AVF patency [14]. Lok et al. reported a median time to maturation of 46 days, slightly longer than observed in our study [15]. These discrepancies may result from differences in the operational definitions of maturation and cannulation criteria. Flow-based findings in our study are also consistent with those of Robbin et al., who noted that a flow rate >500 mL/min at four weeks indicates successful maturation [16]. However, our study did not find early flow rates (day 1 or 7) predictive, possibly due to methodological differences in flow measurement or patient variability.

Primary failure, defined as the failure of the AVF to mature adequately for dialysis use, occurred in 24.8% of cases, which aligns with existing literature reporting failure rates between 20% and 50% [17]. Our analysis revealed a statistically significant association between primary failure and comorbidities, including diabetes (p = 0.05), hypertension (p = 0.002), and their combination (p < 0.001). These findings support those of Bashar et al., who demonstrated that vascular calcification, endothelial dysfunction, and reduced vessel compliance in patients with diabetes and hypertension impair AVF maturation [12]. At four weeks, the maturation rate in our study was 51.4%, which is lower than the 60-70% reported in some previous studies. This discrepancy may be explained by the higher prevalence of diabetes and hypertension in our cohort, as well as the use of stricter criteria for defining maturation. The time to maturation and first cannulation were significantly longer in the primary failure group (42.7 ± 1.4 days and 56.5 ± 2.5 days, respectively) than in those with successful AVFs (32.6 ± 6.4 days and 43.4 ± 6.6 days, respectively; p < 0.001). This highlights the need for early identification and intervention in AVFs at risk of delayed maturation. Our findings regarding the predictive value of preoperative vascular measurements align with those of Lauvao et al., who reported that vein diameters ≥2.5 mm and artery diameters ≥2 mm were associated with successful AVF maturation [18]. Similarly, Wong et al. found that a vein diameter <1.6 mm increased the risk of failure threefold [19].

The most frequent complications observed were thrombosis (9.17%), infection (5.5%), and stenosis (1.83%). No cases of steal syndrome were reported. Complications were significantly more common among patients with failed AVFs: thrombosis occurred in 25.9% vs. 3.6% (p < 0.001), stenosis in 7.4% vs. 0% (p = 0.01), and infection in 14.8% vs. 2.4% (p = 0.01). These findings are consistent with those of Lee et al., who identified thrombosis as the most common cause of AVF failure [20]. The complication rates observed are comparable to those reported by Al-Jaishi et al., who found thrombosis and infection rates of 12.3% and 4.5%, respectively, in a systematic review [21]. The higher rate of complications in the primary failure group underscores the interrelationship between inadequate maturation and adverse outcomes.

Limitations

This study has several significant limitations that should be acknowledged. First, the single-center design may limit the generalizability of our findings. Practice patterns, surgical expertise, and patient characteristics can vary across institutions, potentially influencing outcomes. Future multicenter studies are encouraged to validate our results across broader populations and clinical settings.

Second, while the relatively short follow-up period of four weeks is sufficient for assessing early AVF maturation, it does not capture long-term outcomes such as secondary patency or late-onset complications. A longer follow-up duration would be necessary to assess the durability and clinical success of the fistulas over time.

Third, although we standardized sonographic evaluations and described the surgical approach in detail, inherent surgical technique and operator experience variability may still influence results. These factors could be unmeasured confounders, affecting maturation rates and Doppler findings. Implementing uniform surgical protocols and broader operator training across centers would improve reproducibility and control for these variables in future studies.

Fourth, due to the study's observational nature, our findings represent associations rather than definitive causal relationships. Confounding factors such as intraoperative decision-making, comorbidities, and medication use were not controlled or adjusted for, which may limit the interpretability of some associations.

Finally, while our study used strict maturation criteria aligned with the "Rule of 6" for clinical relevance, these criteria may differ from those used in prior literature, contributing to variability in reported outcomes. This underscores the importance of standardizing outcome definitions in future research.

Despite these limitations, the prospective design and structured ultrasound follow-up provide valuable insights into AVF maturation. Future research should include longer-term follow-up, multicenter validation, and more comprehensive control for surgical and patient-level confounders to build on these findings.

## Conclusions

This study underscores the pivotal role of DUS in assessing and managing AVFs for hemodialysis. Preoperative vascular parameters, particularly cephalic vein and radial artery diameters, were significant predictors of AVF maturation and primary failure. Postoperative flow measurements at four weeks served as valuable prognostic indicators, with lower flow rates strongly associated with failure. As a non-invasive and informative modality, DUS supports clinical decision-making throughout the AVF lifecycle, from preoperative planning and postoperative monitoring to early complication detection. Its integration into routine clinical practice may improve vascular access outcomes and enable personalized care strategies for patients undergoing hemodialysis.
